# Mining definitions in Kissat with Kittens

**DOI:** 10.1007/s10703-023-00421-2

**Published:** 2023-04-24

**Authors:** Mathias Fleury, Armin Biere

**Affiliations:** 1 Institute for Formal Models and Verification, Johannes Kepler University, Linz, Austria; 2 Chair of Computer Architecture, Albert-Ludwigs-University, Freiburg, Germany

**Keywords:** SAT Solving, Variable elimination, Definition extraction

## Abstract

Bounded variable elimination is one of the most important preprocessing techniques in SAT solving. It benefits from discovering functional dependencies in the form of definitions encoded in the CNF. While the common approach pioneered in SatELite relies on syntactic pattern matching, our new approach uses cores produced by an embedded SAT solver, Kitten. In contrast to a similar semantic technique implemented in Lingeling based on BDD algorithms to generate irredundant CNFs, our new approach is able to generate DRAT proofs. We further discuss design choices for our embedded SAT solver Kitten. Experiments with Kissat show the effectiveness of this approach.

## Dedication

We dedicate this rather technical SAT paper to the memory of Ed Clarke. He was one of the first to see the tremendous potential of SAT solving not only in model checking, but more general in verification and beyond. His vision to use SAT for model checking, the encouragement and guidance he gave to two Post-Docs working on this topic (the 2nd author and Yunshan Zhu), which then lead to our multiple awards winning joint work on Bounded Model Checking [5–9, 16], clearly plays a pivotal role in the history of the SAT revolution we are witnessing today.

Bounded Model Checking turned out not only to become the first practical application of SAT but also, even though highly debated initially, lead to a paradigm shift in using formal verification, trading completeness for scalability. This controversy can also be seen as the starting point of other highly-influential work in the model checking community, particularly Ken McMilan’s work on interpolation [30] and then the development of the IC3 algorithm by Aaron Bradley [14], which both also rely on SAT solving but try to keep completeness without sacrifying scalability too much.

This success of SAT in model checking motivated new research on SAT solving, including the seminal work at Princeton yielding the Chaff [32] SAT solver, which is standing on the shoulders of another seminal work around the Grasp solver from Michigan [36], and also turbo-charged the use of decision procedures originating in the automated theorem proving community in the form of SMT. This SAT revolution is a corner stone of the more broader adoption of automated reasoning in many applications, from classical hardware to software verification as well as scheduling cloud jobs. We believe without Ed this would not have happened.

## Introduction

Preprocessing and particularly inprocessing [26] is a key feature of modern SAT solvers, the latter being part of every winner of the SAT competition since 2013. Arguably the most important pre- and inprocessing technique is bounded variable elimination (BVE). Even though in its unbounded form, elimination is a decision procedure for SAT, in the context of preprocessing bounded variable it is not run until completion. The idea of BVE is to iteratively eliminate one variable from the problem by resolving every occurrence away without adding redundant clauses. Furthermore, the difference between the number of added and removed clauses is bounded in practical implementation (Sect. 3).

Definability is a concept that reduces the number of clauses to add. It consists in recognizing a definition of $x$ such that $x \leftrightarrow f(a_1,\dots ,a_n)$ from the input formula in conjunctive normal form (CNF). The simplest example are gates like $x \leftrightarrow a_1\wedge a_2$ that can be efficiently detected. Detecting gates reduces the number of resolvents because not all clauses have to be resolved together. A simple approach is to syntactically recognize gates as encoded in the CNF input. This approach is for example used in CaDiCaL [10] and CryptoMiniSat [39].

This syntactic approach (Sect. 4) is limited though and fails to recognize “irregular” gates not characterized by a simple gate type (such as And gates). It also fails to detect gates after elimination of one of the input variables. Recently semantic approaches based on Padoa’s theorem [34] have been developed with applications in model counting [28] and a similar technique exists for (D)QBF reasoning [35, 37]. In both approaches a SAT solver is used as oracle to find gate clauses. In this paper we follow this line of research and extend our SAT solver Kissat [12] to detect gates semantically. It uses a simple SAT solver called Kitten, called as an oracle to find gate clauses (Sect. 5). Our definition of gate detection is equivalent to previous approaches, even though our method never explicitly reconstructs the function (Sect. 6).

Our technique discovers gates but it does not need to know which are the inputs (Sect. 7). One interesting property about gates is that we do not need to resolve gate clauses among themselves. However, this only holds if the full clause is found and not a subset of the clause. If those clauses are forgotten, an unsatisfiable problem can become satisfiable (Sect. 8). Syntactic detection of gates is faster and detects most useful gates. So Kissat first finds gates syntactically and then calls Kitten to find other gates semantically (Sect. 9).

It turns out that the performance of the sub-solver Kitten has a non-negligible impact on the overall performance, as it is frequently called to find definitions with different environment clauses in which a candidate variable to be eliminated occurs. Basically Kitten is a very simple CDCL solver with watched literals but for instance without blocking literals. A key feature of Kitten for semantic gate detection is that it can be “cleared” efficiently avoiding reallocation of internal data structures (Sect. 10). It further can be instructed to keep antecedents of learned clauses in memory and thus can compute clausal cores in memory.

Experiments on benchmarks from the SAT Competition 2020 show that our new elimination method has only a minor impact on performance and runtime, but it does eliminate substantially more variables, even after syntactic extraction is employed first. Thus definition extraction is effective (Sect. 11).

We finish with related work (Sect. 12). The idea of not generating redundant unnecessary clauses relates to blocked clause elimination (BCE), a simplification technique that can remove clauses. Iser [24] also used a SAT solver in the context of gate identification, but he does not use it to identify a gate, but only to check “right uniqueness” of already identified set of clauses.

This paper is an substantially extended version of our very brief presentation in the system description [12] of Kissat from the SAT Competition 2021 and an extension of our (unpublished) *Pragmatics of SAT Workshop 2021 (POS’21)* presentation [11]. Compared to the system description, we have significantly extended all explanations and give more details about Kitten. Last but not least we report detailed experiments.

## Bounded variable elimination

In principle, eliminating variables from a formula reduces the search space in solving the formula exponentially with the number of removed variables. However, this argument is only sound as long the formula does not increase in size geometrically with the number of eliminated variables. Otherwise we would have found a procedure to polynomially solve SAT.

Thus the basic idea of bounded variable elimination is to only eliminate variables in a formula, for which the resulting formula is not bigger than the original formula, i.e., where the size increase due to variable elimination is bounded. This procedure can be implemented efficiently and in practice is considered the most effective preprocessing technique, particularly for industrial instances.

The basic approach works as follows. Let *x* be a variable considered to be eliminated from the CNF $F$. We split *F* syntactically into three parts

$$\begin{aligned} F \,=\, \underbrace{F_x \wedge F_{{\bar{x}}}}_{E(F,x)} \, \wedge \, \varDelta (F,x), \end{aligned}$$

where $F_\ell$ is the CNF of clauses of *F* which contain literal $\ell$, with $\ell \in \{x, {\bar{x}}\}$ and $\varDelta (F,x)$ contains the remaining clauses without *x* nor ${\bar{x}}$. We call $E(F,x) = (F_x \wedge F_{{\bar{x}}})$ the *environment* of *x*. As usual tautologies do not have to be considered, where a clause is called *tautological* or *trivial* if it contains a variable *x* and its negation ${\bar{x}}$.

Let *x* be a variable and $H_x$ and $H_{{\bar{x}}}$ CNFs where clauses in $H_\ell$ all contain $\ell$, we define the^1^*set of resolvents* of $H_x$ and $H_{{\bar{x}}}$ over *x* as follows:

$$\begin{aligned} H_x \otimes H_{{\bar{x}}} = \{ (C \vee D) \mid (C \vee x) \in H_x,\; (D \vee {\bar{x}}) \in H_{{\bar{x}}} \text{, } \text{ and } (C \vee D) \text{ not } \text{ a } \text{ tautology } \}. \end{aligned}$$

As usual we interpret a CNF also as a set of clauses. The goal of variable elimination is to resolve all clauses of $F_{{\bar{x}}}$ with all clauses of $F_{x}$ and replace *E*(*F*, *x*) with the obtained resolvents, that is replacing the formula *F* by $(F_x \otimes F_{{\bar{x}}}) \wedge \varDelta (F,x)$.

The process described so far is just a reformulation of “clause distribution” from the original DP procedure [17]. What turns it into the most important preprocessing techniques of today’s SAT solvers is the idea of eliminating a variable if the difference between the number of added (resolvent) clauses and removed clauses (containing the eliminated variable *x*) is bounded [2, 3, 18, 40]. There are various possibilities to set this bound, and even increase it dynamically [33], which are orthogonal to the discussion of this paper.

Enforcing that the size of the formula does not grow too much during variable elimination restricts the number of variables that can be eliminated and thus the effectiveness of variable elimination. It is therefore beneficial to determine whether certain resolvents are redundant, i.e., implied by the resulting formula, and do not need to be added. This will allow additional variables to be eliminated, for which the size limit is hit without considering redundant resolvents.

Finally, as the elimination of a variable produces a formula which is satisfiability equivalent but not logically equivalent to the original formula (unless the formula is unsatisfiable), we need a way to reconstruct models of the original formula given a model of the simplified formula. This can be achieved by saving the eliminated clauses on a “reconstruction stack” and the interested reader might want to consult [13, 21, 26] for further details.

## Gate extraction

Already when introducing the SatELite preprocessor [18], it was proposed to extract subsets of “gate clauses” from $F_x$ and $F_{{\bar{x}}}$ that encode “circuit gates” with output *x*, also called *definitions* of *x*. Resolving these gate clauses against each other results in tautological (trivial) resolvents, and, in particular, this situation allows the solver to ignore resolvents between non-gate clauses (since those are implied). Assume that *F* can be decomposed as follows

$$\begin{aligned} F \;\equiv \; \overbrace{ G_x \wedge H_x }^{F_x} {} \wedge {} \overbrace{ G_{{\bar{x}}} \wedge H_{{\bar{x}}} }^{F_{{\bar{x}}}} {} \wedge {} \varDelta (F,x) \end{aligned}$$

where $G \equiv G_x \wedge G_{{\bar{x}}}$ are the *gate clauses*, i.e., the Tseitin encoding of a circuit gate with output *x*, $H_x$ and $H_{{\bar{x}}}$ the remaining *non-gate clauses* of *F* containing *x* and ${\bar{x}}$ respectively, and $\varDelta (F,x)$ the remaining clauses without *x* nor ${\bar{x}}$. The original technique from SatELite [18] would then use

$$\begin{aligned} F \;\equiv \; (F_x \otimes F_{{\bar{x}}}) \wedge \varDelta (F,x) \;\equiv \; (G_x \otimes H_{{\bar{x}}}) \wedge (G_{{\bar{x}}} \otimes H_x) \wedge \varDelta (F,x) \end{aligned}$$

and only consider the smaller set of resolvents on the right, as both $G_x \otimes G_{{\bar{x}}}$ as well $H_x \otimes H_{{\bar{x}}}$ can be omitted from $F_x \otimes F_{{\bar{x}}}$, even though the former are tautological resolvents and thus ignored anyhow. To give a concrete example consider the following formula containing three gate clauses, encoding an And gate $x = a \wedge b$, and four non-gate clauses.

$$\begin{aligned} F = \underbrace{ ({\bar{a}} \vee {\bar{b}} \vee x) }_{G_x} \mathop {\wedge }\underbrace{ (a \vee {\bar{x}}) \mathop {\wedge }(b \vee {\bar{x}}) }_{G_{{\bar{x}}}} \mathop {\wedge }\overbrace{ (c \vee x) \mathop {\wedge }(d \vee x) }^{H_x} \mathop {\wedge }\overbrace{ (e \vee {\bar{x}}) \mathop {\wedge }(f \vee {\bar{x}}) }^{H_{{\bar{x}}}} \mathop {\wedge }\underbrace{({\bar{c}} \vee {\bar{d}} \vee {\bar{e}} \vee \bar{f})}_{\varDelta (F,x)} \end{aligned}$$

Resolving all clauses with *x* or ${\bar{x}}$ results in the following CNF.
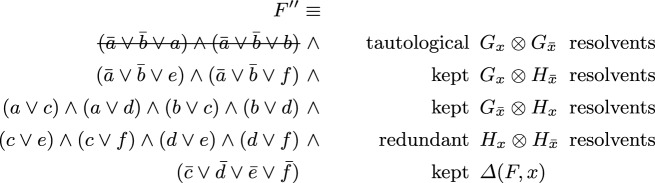


Eliminating *x* in the original CNF *F* of 8 clauses results in CNF $F''$ with 13 clauses in total, but includes 2 tautological clauses, thus actually only has 11 non-tautological clauses. Without further ignoring the 4 redundant resolvents in $H_x \otimes H_{{\bar{x}}}$ bounded variable elimination (even up to allowing for introducing two more clauses) would still not eliminate *x*. If the And gate is detected and non-gate clauses are not resolved against non-gate clauses, we end up with 7 clauses and *x* is eliminated.

Finding such gate clauses was originally based on syntactic pattern matching, by in essence trying to invert the Tseitin encoding. This is best explained for And gates. Given an elimination candidate *x* and $\ell \in \{x, {\bar{x}}\}$. We go over all “base clauses” $C = (\ell \vee \ell _1 \vee \cdots \vee \ell _n)$ and check whether *F* also contains all $({{\bar{\ell }}} \vee {{\bar{\ell }}}_i)$ for $i=1\ldots n$. If this is the case, we found the *n*-ary And gate $\ell = ({{\bar{\ell }}}_1 \wedge \cdots \wedge \bar{\ell }_n)$ with gate clauses $G_\ell = \{ C \}$ and $G_{{{\bar{\ell }}}} = \{ ({{\bar{\ell }}} \vee {{\bar{\ell }}}_i) \mid i=1\ldots n \}$. If $\ell = x$ then *x* is the output of an And gate. If $\ell = {\bar{x}}$, then *x* is the output of an Or gate $x = (\ell _1 \vee \cdots \vee \ell _n)$. For the special case $n=1$ this amounts to extracting bi-implications (equivalences). According to our benchmarks (Sect. 11), extracting And gates this way already gives the largest benefit but similar syntactical extraction techniques exist for Xor or IfThenElse gates.

Detecting gates syntactically, however, is not very robust and our SAT solver Lingeling [4] implements a very different technique inspired by BDD algorithms. It converts the environment clauses into a BDD (actually a function table), eliminates variables there, and translates the result back to a CNF using Minato’s algorithm [19, 31], which produces a redundancy-free CNF. More details are provided in the preprocessing chapter of the 2nd edition of the Handbook of SAT [13].

**Fig. 1:**
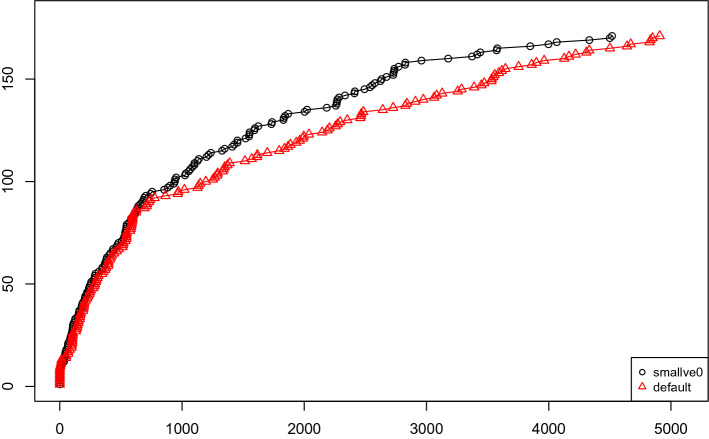
Lingeling with and without variable elimination

**Fig. 2:**
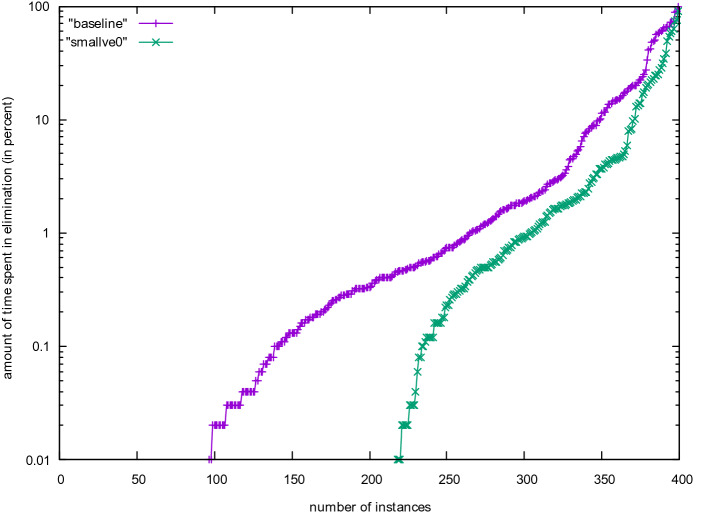
Percentage of the total amount of time spent in variable elimination in Lingeling

Figure 1 shows a CDF of the number of solved instances of the last Lingeling release with and without this technique. On these problems from the SAT Competition 2020, deactivating this technique (smallve0) gives better performance. Remember that Lingeling is not developed anymore and was not trained on competition problems since 2016. Figure 2 gives the amount of time spent during variable elimination. As Lingeling’s semantic variable elimination algorithm is arguably too costly, we take this as an additional motivation to look into different algorithms for semantic gate detection. The second issue with the implementation is that it cannot produce a DRAT proof of the transformation.

## Definition mining with a SAT solver

Instead of only syntactically extracting definitions, our new version of Kissat tries to extract gate clauses semantically by checking satisfiability of the conjunction of the co-factors $(F_x|{}_{{\bar{x}}})$ and $(F_{{\bar{x}}}|{}_x)$ of *F*, i.e., the formula that is obtained by removing the occurrences of *x* in $F_x$ and of ${\bar{x}}$ in $F_{{\bar{x}}}$ and then conjoining the result. Alternatively one can obtain the candidate formula to be checked for unsatisfiability by removing all occurrences of the literals *x* and ${\bar{x}}$ from the environment *E*(*F*, *x*).

If this formula is unsatisfiable, we compute a clausal core which in turn can be mapped back to original gate clauses $G_x$ and $G_{\bar{x}}$ in the environment (by adding back *x* resp. ${\bar{x}}$ to the clauses generated in the first step).

Note that we ignore $\varDelta (F,x)$ here and focus on environment clauses only. In principle, however, we can replace $\varDelta (F,x)$ in *F* by $(x \vee \varDelta (F,x)) \wedge ({\bar{x}} \vee \varDelta (F,x))$ to obtain a CNF (after distributing the variables over $\varDelta (F,x)$) where all clauses either contain *x* or ${\bar{x}}$. Thus the following discussion extends to the seemingly more general case where also $\varDelta (F,x)$ is used as “don’t care” for gate extraction.

Let $G_\ell$ for $\ell \in \{x, {\bar{x}}\}$ be the identified clauses of $F_\ell$ mapped back from the clausal core computed by the SAT solver and $H_\ell$ the remaining clauses, i.e., $F_\ell = G_\ell \wedge H_\ell$. Then it turns out that $F_x \otimes F_{{\bar{x}}}$ can be reduced to $(G_x \otimes G_{{\bar{x}}}) \wedge (G_x \otimes H_{{\bar{x}}}) \wedge (G_{{\bar{x}}} \otimes H_x)$. In particular $(H_x \otimes H_{{\bar{x}}})$ can be omitted.^2^ The net effect is that fewer resolvents are generated and thus more variables can be eliminated.

To see that non-gate versus non-gate resolvents can be omitted assume that $A \wedge B$ is unsatisfiable and thus ${\bar{A}} \vee \bar{B}$ is valid. Therefore for any *C* or *D* we have

$$\begin{aligned} (A \vee C) \wedge (B \vee D) \;\equiv \; (A \vee C) \wedge (B \vee D) \wedge ({\bar{A}} \vee {\bar{B}}). \end{aligned}$$

With two resolution steps we can then show that the right-hand side implies $(C \vee D)$ and thus can be added to the left-hand side.

$$\begin{aligned} (A \vee C) \wedge (B \vee D) \;\equiv \; (A \vee C) \wedge (B \vee D) \wedge (C \vee D) \end{aligned}$$

Setting $(A,B,C,D) = (G_x|{}_{{\bar{x}}},G_{{\bar{x}}}|{}_x,H_{\bar{x}}|{}_x,H_x|{}_{{\bar{x}}})$ shows the rest, more specifically, that $C\vee D = H_{{\bar{x}}}\vee H_x|{}_{{\bar{x}}}$ can be ignored, independent of $A \vee B = G_x|{}_{{\bar{x}}},G_{{\bar{x}}}|{}_x$:

$$\begin{aligned} F_x \otimes F_{{\bar{x}}}\equiv & {} (G_x \otimes G_{{\bar{x}}}) \wedge (G_x \otimes H_{{\bar{x}}}) \wedge (G_{{\bar{x}}} \otimes H_x) \wedge (H_x \otimes H_{{\bar{x}}}) \\\equiv & {} (G_x|{}_{{\bar{x}}} \vee G_{{\bar{x}}}|{}_{x}) \wedge (G_x|{}_{{\bar{x}}} \vee H_{{\bar{x}}}|{}_{x}) \wedge (G_{{\bar{x}}}|{}_{x} \vee H_x|{}_{{\bar{x}}}) \wedge (H_x|{}_{{\bar{x}}} \vee H_{\bar{x}}|{}_{x}) \\= & {} (A \vee B) \wedge (A \vee C) \wedge (B \vee D) \wedge (C \vee D) \\\equiv & {} (A \vee B) \wedge (A \vee C) \wedge (B \vee D) \\= & {} (G_x \otimes G_{{\bar{x}}}) \wedge (G_x \otimes H_{{\bar{x}}}) \wedge (G_{{\bar{x}}} \otimes H_x) \end{aligned}$$

For the previous example the conjunction of the co-factors of the 7 environment clauses *E*(*F*, *x*) results in the following unsatisfiable formula

$$\begin{aligned} ({\bar{a}} \vee {\bar{b}}) \wedge (a) \wedge (b) \wedge (c) \wedge (d) \wedge (e) \wedge (f). \end{aligned}$$

The first three clauses form a clausal core and after adding back *x* and ${\bar{x}}$ enable extracting the same gate clauses as before, which in turn enables bounded variable elimination. If only one co-factor contains clauses, e.g., $H_{{\bar{x}}}$, then we can learn the unit literal $x$. This rarely happens in our experiments though. This technique is a generalization of failed literal probing [29] where multiple decisions are allowed instead of deciding and propagating just one literal.

## Relating functional dependency and cores

In previous work [28, 34, 37] the following condition for “definability” was used and we are going to show that in essence it boils down to the same idea. A variable *x* has a *functional dependency* in *F* on an (ordered) sub-set of variables *D* of *F* with $x \not \in D$, i.e., the set *D* of other variables on which the value of *x* is functionally dependent, iff the following formula is valid

1 $$\begin{aligned} (D = D') \wedge F \wedge F' \;\rightarrow \; x = x' \end{aligned}$$

with $F'$ a copy of *F* where each variable *y* is replaced by a new variable $y'$. The intuitive meaning is that there is only one solution for $x$ given the same inputs ($D=D'$), whatever the value of the other variables.

The short-hands $D = D'$ and $x = x'$ denote formulas which enforce that the corresponding original variable and its primed copy assume the same value (through for instance a conjunction of bi-implications). Therefore, there is a functional dependency of *x* on *D* iff the following formula is unsatisfiable.

$$\begin{aligned} (D = D') \wedge F \wedge F' \wedge ({\bar{x}} = x') \end{aligned}$$

The key remark is that ${\bar{x}} = x'$ and $\overline{x=x'}$ are equivalent because the formula is symmetric in $x$ and $x'$. In our concrete application, we are not interested in determining the exact set of variables $D$, because we do not have restrictions on dependencies (unlike in QBF [37] or #SAT [28]). Hence we can pick $D$, i.e., the variables on which *x* is supposed to depend, to consist of an arbitrary set of variables occurring in $F$ except $x$. In practice we will restrict *D* to the set of variables in the environment of *E*(*F*, *x*) different from *x* and this way obtain a sufficient but not necessary condition for definability of *x* over *F*.

Under this assumption, we prove that our core based condition is the same as definability. First determine CNFs *P*, *N* and *R* such that

$$\begin{aligned} F \;\equiv \; (x \vee P) \wedge ({\bar{x}} \vee N) \wedge R \end{aligned}$$

where neither *x* nor ${\bar{x}}$ occurs in *R*. Then simplify $(D = D') \wedge F' \wedge ({\bar{x}} = x')$ to

$$\begin{aligned} \begin{array}{rcl} (F \wedge F') [D' \mapsto D] [x' \mapsto {\bar{x}}] &{}=&{} F \wedge (F' [D' \mapsto D] [x' \mapsto {\bar{x}}]) \\ &{}=&{} F \wedge \left( \left( (x' \vee P') \wedge (x' \vee N') \wedge R'\right) [D' \mapsto D] [x' \mapsto {\bar{x}}]\right) \\ &{}=&{} F \wedge \left( \left( (x' \vee P) \wedge (x' \vee N) \wedge R\right) [x' \mapsto {\bar{x}}]\right) \\ &{}=&{} F \wedge \left( ({\bar{x}} \vee P) \wedge (x \vee N) \wedge R\right) \end{array} \end{aligned}$$

using equivalent literal substitution (see for instance [13]). This yields the following satisfiability equivalent formula to our core condition in Eqn. (1)

$$\begin{aligned} F \wedge (F[x \mapsto {\bar{x}}]), \end{aligned}$$

where on the right *x* is replaced by its negation ${\bar{x}}$ and accordingly ${\bar{x}}$ with *x*. As *F* is a CNF this formula contains each clause with *x* twice, once as in *F* and once with *x* (and ${\bar{x}}$) negated. These two copies of each clause can thus be resolved on *x* and each resolvent subsumes both antecedents (through self-subsuming resolution). Clauses in $F'$ which do not contain $x'$ nor ${\bar{x}}'$ become identical after substitution to their counterpart in *F*.

Therefore the resulting formula after substitution is logically equivalent to the formula obtained from *F* by removing all the environment clauses *E*(*F*, *x*) (clauses with *x* or ${\bar{x}}$) and replacing them with $(F_x|{}_{{\bar{x}}}) \wedge (F_{{\bar{x}}}|{}_x)$.

To summarize, in order to determine that *x* is dependent on the variables *D* in *E*(*F*, *x*) it is sufficient to check unsatisfiability of

$$\begin{aligned} (F_x|{}_{{\bar{x}}}) \wedge (F_{{\bar{x}}}|{}_x) \wedge \varDelta (F,x) \end{aligned}$$

### Example 1

(Example of the Proof) Consider the following formula and apply the proof described above: $F = \underbrace{ ({\bar{a}} \vee {\bar{b}} \vee x) }_{G_x} {} \wedge {} \underbrace{ (a \vee {\bar{x}}) \wedge (b \vee {\bar{x}}) }_{G_{{\bar{x}}}}$ as defined above. The formula

$$\begin{aligned} (D = D')&\qquad \quad (a=a' \wedge b=b' \wedge c=c')\\ {}\wedge {} F&\qquad \quad (({\bar{a}} \vee {\bar{b}} \vee x)\wedge (a \vee {\bar{x}}) \wedge (b \vee {\bar{x}})\wedge (c\vee x))\\ {}\wedge {} F'&\qquad \quad (({\bar{a}}' \vee {\bar{b}}' \vee x')\wedge (a' \vee {\bar{x}}') \wedge (b' \vee {\bar{x}}')\wedge (c'\vee x'))\\ \;\rightarrow \; x = x'&\quad \end{aligned}$$

is satisfiable iff its negation is unsatisfiable

$$\begin{aligned} (D = D')&\qquad \quad (a=a' \wedge b=b' \wedge c=c')\\ {}\wedge {} F&\qquad \quad (({\bar{a}} \vee {\bar{b}} \vee x)\wedge (a \vee {\bar{x}}) \wedge (b \vee {\bar{x}})\wedge (c\vee x))\\ {}\wedge {} F'&\qquad \quad (({\bar{a}}' \vee {\bar{b}}' \vee x')\wedge (a' \vee {\bar{x}}') \wedge (b' \vee {\bar{x}}')\wedge (c'\vee x'))\\ {}\wedge \overline{x = x'}&\quad \\ \end{aligned}$$

as the formula is symmetrical in $x$ and $x'$, is unsatisfiable iff the following is too

$$\begin{aligned} (D = D')&\qquad \quad (a=a' \wedge b=b' \wedge c=c')\\ {}\wedge {} F&\qquad \quad (({\bar{a}} \vee {\bar{b}} \vee x)\wedge (a \vee {\bar{x}}) \wedge (b \vee {\bar{x}})\wedge (c\vee x))\\ {}\wedge {} F'&\qquad \quad (({\bar{a}}' \vee {\bar{b}}' \vee x')\wedge (a' \vee {\bar{x}}') \wedge (b' \vee {\bar{x}}')\wedge (c'\vee x'))\\ {}\wedge {\bar{x}} = x'&\quad \end{aligned}$$

We replace equivalent variables:

$$\begin{aligned} F&\qquad \quad (({\bar{a}} \vee {\bar{b}} \vee x)\wedge (a \vee {\bar{x}}) \wedge (b \vee {\bar{x}})\wedge (c\vee x))\\ {}\wedge {} F'[x'\mapsto {\bar{x}}]& (({\bar{a}} \vee {\bar{b}} \vee {\bar{x}})\wedge (a \vee x) \wedge (b \vee x)\wedge (c\vee {\bar{x}})) \end{aligned}$$

Now we resolve each clause of $F$ with its $F'$ counterpart, yielding a clause subsuming its antecedents

$$\begin{aligned}&(({\bar{a}} \vee {\bar{b}})\wedge (a) \wedge (b) \wedge (c)) \end{aligned}$$

and we can use Kitten to determine that these clauses are unsatisfiable and to produce the following clausal core

$$\begin{aligned}&(\bar{a} \vee {\bar{b}})\wedge (a) \wedge (b) \end{aligned}$$

In our approach we focus on the environment $E(F,x) \subseteq F$ and only extract definitions implied by *E*(*F*, *x*), which reduces the effort spent in Kitten, but in principle we might want to take additional clauses of *F* or all of $\varDelta (F,x)$ into account to find all definitions (see Example 2 below). We further do not need a conjecture about *D* a-priori, actually do not even need to determine *D* for our application at all. It is sufficient to extract gate clauses from the proof of unsatisfiability. Their variables make up *D* (excluding *x*).

### Example 2

(Missing Environment) Our extraction without additional clauses can miss definitions. Consider for example, the circuit corresponding to $x\mathbin =a \mathrel \wedge a\mathbin =b$, where we add $b$ (resp. ${\bar{b}}$) to each clause containing $x$ (resp. ${\bar{x}}$) and are looking for the definition of $x$. The CNF is $F = ({\bar{x}}\vee a\vee b) \mathrel \wedge (x\vee {\bar{a}}\vee {\bar{b}})\mathrel \wedge ({\bar{a}} \vee b)\mathrel \wedge (a\vee {\bar{b}})$. Obviously from $F$, we know that $x=a$ or $x=b$ are both definitions of $x$.

$$\begin{aligned} F_{x}|{}_{x} \wedge \, F_{{\bar{x}}}{}|{}_{{\bar{x}}}&= (a\vee b) \mathrel \wedge ({\bar{a}}\vee {\bar{b}})\\ \varDelta (F,x)&=({\bar{a}} \vee b)\mathrel \wedge (a\vee {\bar{b}}) \end{aligned}$$

Without the additional two clauses in $\varDelta (F,x)$, the problem is satisfiable, but becomes unsatisfiable with them. Therefore, our approach without all clauses would miss definability. Remark that in this case, we would actually be able to find the definition of $x$ by first deriving the definition $a$ and eliminating it.

## Actually determining the definition

In order to apply gate information to variable elimination we do not need to extract the actual gate *f*(*D*) of *x* nor need to know the set of input variables *D* of the gate *f*. For other applications it might still be interesting to characterize the possibilities of picking *f* though. Let $L = G |{}_x$ be the positive co-factor of the gate clauses *G* and $U = \overline{G|{}_{{\bar{x}}}}$ the negation of its negative co-factor, where, to simplify the argument, we use $G_x|{}_x = G_{{\bar{x}}} | {}_{{\bar{x}}} = \top$, and thus

$$\begin{aligned} G|{}_x \;\equiv \; (G_x \wedge G_{{\bar{x}}})|{}_x \;\equiv \; G_x|{}_x \wedge G_{{\bar{x}}}|{}_x \;\equiv \; G_{{\bar{x}}}|{}_x \;\equiv \; L \end{aligned}$$

and

$$\begin{aligned} G|{}_{{\bar{x}}} \;\equiv \; (G_x \wedge G_{{\bar{x}}})|{}_{{\bar{x}}} \;\equiv \; G_ x|{}_{{\bar{x}}} \wedge G_x|{}_{{\bar{x}}} \;\equiv \; G_x|{}_{{\bar{x}}} \;\equiv \; {\overline{U}}. \end{aligned}$$

This notation allows us to derive the following “Shannon decomposition” of *G*:

$$\begin{aligned} G \;\equiv \; ({\bar{x}} \,\vee \, G|{}_x) \wedge (x \,\vee \, G|{}_{\bar{x}}) \;\equiv \; ({\bar{x}} \,\vee \, G_{{\bar{x}}}|{}_x) \wedge (x \,\vee \, G_x|{}_{{\bar{x}}}) \;\equiv \; ({\bar{x}} \vee L) \wedge (x \vee \overline{U}) \end{aligned}$$

First note that *L* implies *U* (written $L \models U$) as $L \wedge {\overline{U}}$ is the same as $G_{{\bar{x}}}|{}_{x} \wedge G_x|{}_{\bar{x}}$ and thus unsatisfiable. Now pick an arbitrary *f* with $L \le f \le U$ between the lower bound *L* and the upper bound *U*, i.e., $L \models f$ and $f \models U$. We are going to show that $G \models x = f$.

The lower bound gives ${\bar{x}} \vee L \models {\bar{x}} \vee f$ and as $G \models {\bar{x}} \vee L$ we get $G \models {\bar{x}} \vee f$ by modus ponens. Similarly we have $x \vee {\overline{U}} \models x \vee \bar{f}$ by contraposition of the upper bound assumption, i.e., $\overline{U} \models {\bar{f}}$, and derive $G \models x \vee {\bar{f}}$, which concludes the proof. If *f* is given explicitly we can pick *D* as the set of variables occurring in *f*. If *f* is given semantically, for instance as function table or BDD, then $y \in D$ iff $f|{}_y \not \equiv f|{}_{{\bar{y}}}$, which can be determined by checking equivalence between co-factors. Similar arguments can be used for characterizing gate extraction from BDDs [20, 41].

## Resolving gate against gate clauses

As we have explained above the idea of gate extraction is that we only need to resolve clauses with the definition of the gate. However, we still need to resolve the gate clauses amongst themselves in two cases. First if extracted semantically (Sect. 8.1). Second if instead of finding a clause, we actually find a *shorter* (subsuming) clause (Sect. 8.2). Both cases are easy to detect in an implementation.

### Semantical gate extraction

Semantic definition extraction does not necessarily produce gate clauses which are tautological, i.e., $G_x \otimes G_{{\bar{x}}}$ could be non-empty. If these resolvents among gate clauses are not added to the clause set, variable elimination is not satisfiability preserving. Consider the following (unsatisfiable) formula:

$$\begin{aligned} F \;=\; \underbrace{ (x \vee b) }_{G_x} {} \wedge \underbrace{ (\bar{x} \vee a) \wedge ({\bar{x}} \vee {\bar{a}} \vee {\bar{b}}) }_{G_{{\bar{x}}}} {} \wedge \underbrace{ (x \vee {\bar{a}}) }_{H_x} {} \wedge {} \overbrace{ ({\bar{a}} \vee c) \wedge (a \vee {\bar{b}}) \wedge (b \vee {\bar{c}}) }^{\varDelta (F,x)} \end{aligned}$$

As shown, Kitten found the (actually minimum unsatisfiable) clausal core $(b) \wedge (a) \wedge ({\bar{a}} \vee {\bar{b}})$ in the conjunction of the co-factors of the environment of *x*, even though there is a shorter core $(a) \wedge ({\bar{a}})$, which after adding back ${\bar{x}}$ and *x* encodes a bi-implication. The reader should be aware that the extracted gate clauses do not encode a Nand gate (second clause has ${\bar{x}}$ and not *x*).

This example was produced through fuzzing [15], by comparing a version of Kissat which correctly resolves gate clauses and one which does not. In this example the fuzzer produced an option setting where extraction of equivalences (bi-implications) was disabled before semantic definition extraction was tried, and then Kitten simply focused on the larger core.
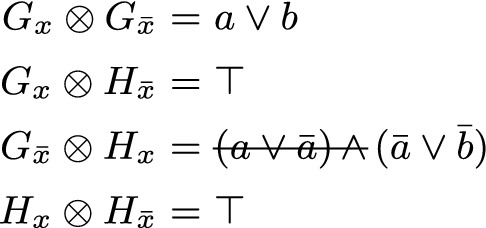


Thus the correct result after elimination is

$$\begin{aligned} F'' \;=\; \underbrace{ (a \vee b) }_{G_x \otimes \,G_{{\bar{x}}}} {} \wedge {} \underbrace{ ({\bar{a}} \vee {\bar{b}}) }_{G_{{\bar{x}}} \otimes \,H_x} {} \wedge \overbrace{ ({\bar{a}} \vee c) \wedge (a \vee {\bar{b}}) \wedge (b \vee {\bar{c}}) }^{\varDelta (F,x)}. \end{aligned}$$

The last four clauses are satisfiable (setting $a = b = c = \bot$) but the whole $F''$ as *F* is unsatisfiable. Therefore the first clause obtained from resolving gate with gate clauses has to be added.

### Syntactical gate resolving

We have used fuzzing again to show that the requirement to add gate against gate resolvents is not unique to semantic gate extraction, but also applies to syntactic gate extraction if for instance one allows the solver to use shorter subsuming clauses instead of the exact Tseitin clauses (a common case in Xor extraction [38]). Consider the following encoding of “$x = (\textrm{if}~a~\textrm{then}~b~\textrm{else}~c)$”, encoded as:

$$\begin{aligned} \begin{array}{rcl} G_{x} &{}=&{}(x\vee {\bar{a}}\vee {\bar{b}}) \wedge (x\vee a\vee {\bar{c}})\\ G_{{\bar{x}}}&{}=&{} ({\bar{x}}\vee c) \wedge ({\bar{x}}\vee {\bar{a}}\vee b)\\ F' &{}=&{} (b\vee {\bar{a}}\vee c) \wedge (a\vee c) \wedge (a\vee {\bar{c}}) \wedge ({\bar{a}}\vee {\bar{c}}) \end{array} \end{aligned}$$

By resolving on $x$, we obtain:

$$\begin{aligned} \begin{array}{rcl} G_x \otimes G_{{\bar{x}}} &{}=&{} ({\bar{a}}\vee {\bar{b}}\vee c) \wedge (a \vee {\bar{b}}\vee {\bar{c}}) \\ G_x \otimes H_{{\bar{x}}} &{}=&{} \top \\ G_{{\bar{x}}} \otimes H_x &{}=&{} \top \\ H_x \otimes H_{{\bar{x}}} &{}=&{} \top \end{array} \end{aligned}$$

If we do not include the resolvents, then $b$ actually becomes pure and the entire formula is satisfiable with $a=\bot$ and $b = c = \top$. However the formula is actually unsatisfiable. The resolvent of $G_{x}\otimes G_{{\bar{x}}}$ contains the clause ${\bar{a}}\vee {\bar{b}}\vee c$. By resolving with the first clause $b\vee {\bar{a}}\vee c$ of $F'$, we obtain the clause ${\bar{a}}\vee c$ meaning that the clauses are unsatisfiable, because we now have all binary clauses over $a$ and $c$.

## Scheduling variable in the main SAT solver Kissat

Identifying gate clauses syntactically is more efficient than identifying UNSAT cores with a SAT solver, even when using a smaller one like Kitten. Hence, Kissat first uses syntactic pattern matching for a Tseitin encoding of an And, Equivalence, Xor, or IfThenElse gate with the given variable as output, and only if this fails, the inner SAT solver is called. In turn, if this fails due to hitting some limits, the standard elimination criterion is used. This is illustrated in Algorithm 1.

**Fig. 3:**
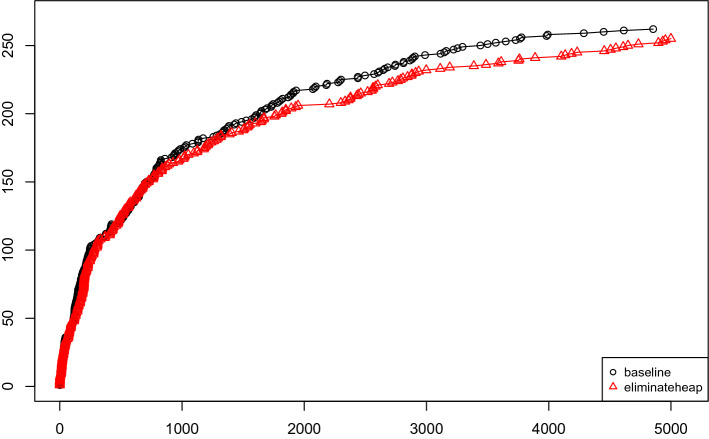
Performance of Kissat with and without heap to schedule variable elimination

Until 2020, the order of scheduling variables as candidates to be eliminated was done using a priority queue implemented as binary heap, where variables with smaller number of occurrences are tried to be eliminated first. Since the 2021 version, we have (by default) disabled the heap and replaced it with iterating over all active literals; i.e., the variables that have neither been removed nor have already been eliminated. This actually improves performance of Kissat (Fig. 3). Of course it avoids updating the heap when removing clauses and probably has other positive effects we still need to investigate in future work.
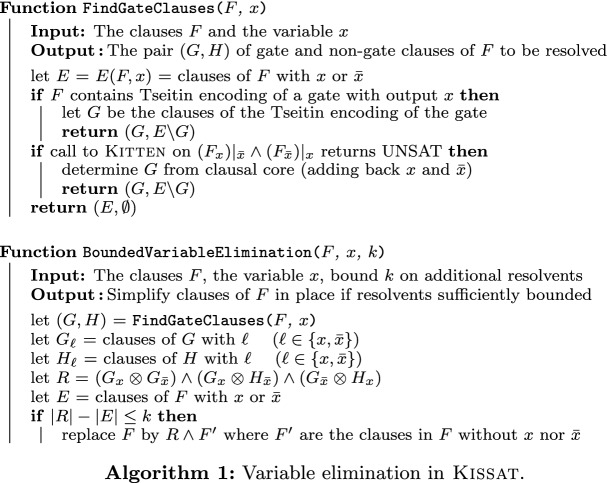

## Core-producing lean embedded SAT solver Kitten

In order to check satisfiability and compute clausal cores of these co-factors of the environment of a variable we have implemented a simple embedded sub-solver Kitten with in-memory proof tracing and fast allocation and deallocation. If the conjunction of the co-factors of the environment are unsatisfiable we reduce through the API in Kitten its formula to the clausal core, shuffle clauses and run Kitten a second time which usually results in a smaller core and thus fewer gate clauses (increasing chances that the variable is eliminated).

If only one co-factor contains core clauses, then we can derive a unit clause. In this case the learned clauses in Kitten are traversed to produce a DRAT proof trace sequence for this unit. This is one benefit of using a proof tracing sub-solver in contrast to the BDD inspired approach in Lingeling [4] discussed at the end of Sect. 4, which cannot produce DRAT proofs.

Kitten is a very simple SAT solver. Instead of using complicated data structures that take a long time to initialize, Kitten uses watched literals (without blocking literals) and the variable-move-to-front heuristic for decisions. It does not feature garbage collection (no “reduce”) nor simplification of added unit clauses. The latter makes it easier to keep track of unsat cores.

To speed up solving and reduce memory usage, Kitten renumbers literals of the given clauses to consecutive literals. Allocations are very fast reusing the internal memory allocator of Kissat instead of allocating new memory. However, even though allocation is fast, it is better to *reuse* the space allocated Kitten within one elimination round. In order to reuse Kitten for the next variable we only clear the necessary content of memory, by for instance clearing stacks for watch lists and the clause arena, instead of deleting and reallocating the solver.

## Experiments

We have evaluated Kissat on the benchmark instances from the SAT Competition 2020 on 8-core Intel Xeon E5-2620 v4 CPUs running at 2.10GHz (turbo-mode disabled). We used a memory limit of 7GB (unlike the SAT Competition 2020).

**Fig. 4:**
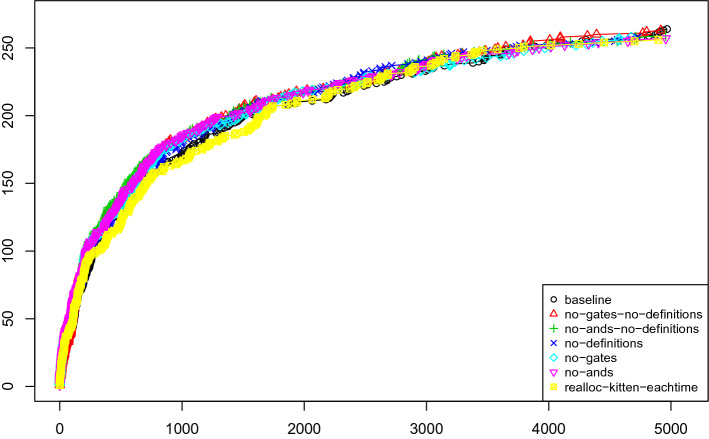
Kissat with various options of gates and definitions in variable elimination

In our first experiment, we have run Kissat with and without gates for variable elimination. The results are presented in Fig. 4 and the difference is rather negligible. While the default version performs slightly better, the difference is too small to be significant. However performance is also not worse. The graph also includes the configuration realloc-kitten-eachtime where instead of clearing and reusing the same Kitten instance during elimination rounds, Kissat reallocates a new Kitten solver for each variable. Thus avoiding this reallocation turns out to be important at the beginning, even if the impact seems to wear off over time.

**Fig. 5:**
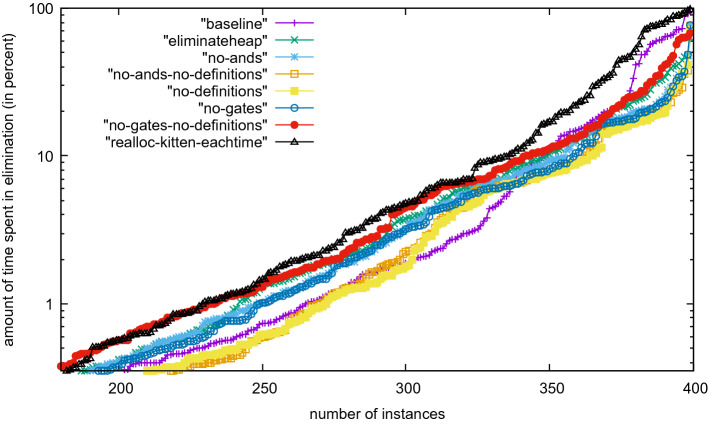
Percentage of time spent in variable elimination and gate extraction relative to the overall individual running time per benchmark for all 400 SAT Competition 2020 main track instances with time limit 5000 s, including benchmarks for which the various versions timed-out. The 100% upper bound on the *y*-axis reached for some instances means that all time was spent in variable elimination

We also plotted the amount of time used in the entire elimination procedure (not only the time spent in Kitten). Figure 5 shows that the time spent in Kitten is similar for most problems but in extreme cases is much larger even though the effect is not critical most of the time. However, if we activate preprocessing as described in the next paragraph, we observed extreme cases (like newpol34-4) where the elimination took more than 90% of the time. However, these problems are not solved by any Kissat configuration anyhow.

**Fig. 6:**
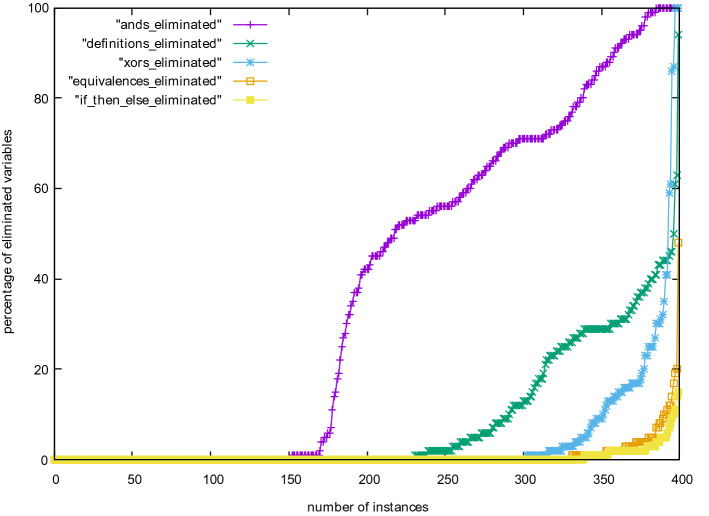
Percentage of variables eliminated relative to the overall number of eliminated variables of individual benchmarks for all 400 SAT Competition 2020 main track instances, with time limit 5000 s, including benchmarks, for which the various versions timed-out. The upper bound 100% on the *y*-axis reached by some instances means that for all eliminated variables we found (syntactic or semantic) gates and used these during elimination

**Fig. 7:**
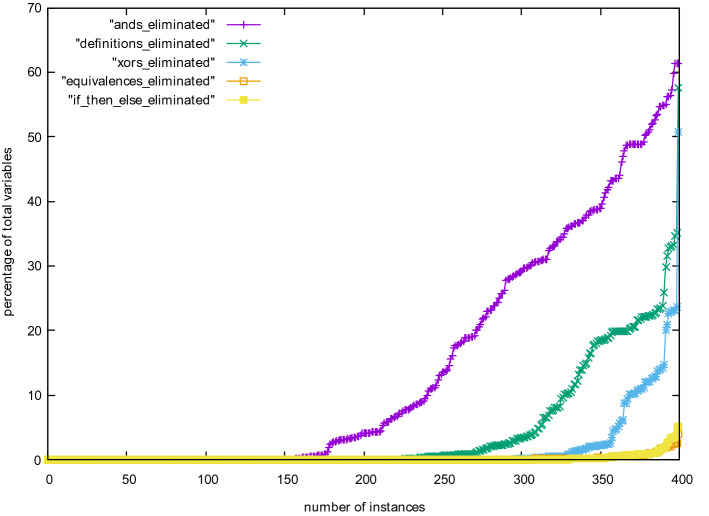
Percentage of variables eliminated relative to the overall *total* number of variables of individual benchmarks for all 400 SAT Competition 2020 main track instances, with time limit 5000 s, including benchmarks, for which the various versions timed-out. The maximum 61% on the *y*-axis reached by some instances means that 61% of all variables in the input problem have been eliminated by detecting the given gate (syntactic or semantic) during elimination

We have further compared efficiency of different techniques by looking at how many variables they have eliminated compared to the total number of eliminated variables (Fig. 6). We can see that And-gate elimination is by far the most important, but semantically extracting definitions is second. Extracting IfThenElse gates is not essential. Still, for all extraction techniques, there are a few problems where nearly all eliminated variables are of the given type. We assume that this is due to the structure and the encoding of those problems. Figure 7 shows the same numbers in relation to the total number of variables of the input problem and not compared to the number of eliminated variables, with the same conclusion: And-gate elimination is more important than any other technique.

To evaluate our new elimination technique in more detail, we implemented a preprocessing phase in Kissat, by running explicit preprocessing rounds initially. Each round is composed of probing, vivification, and variable elimination. For our experiments, we use three rounds of preprocessing (or fewer if a fix-point is reached earlier). Then we do not run Kissat until completion and stop at the first decision. In the default implementation, there is no preprocessing and the same techniques are only called as inprocessing after a few hundred conflicts.

**Fig. 8:**
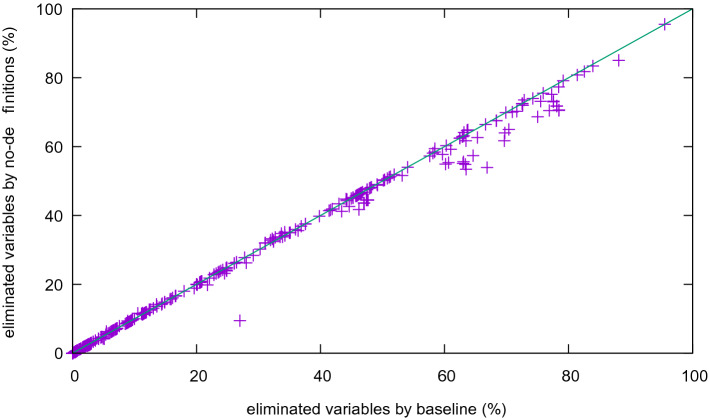
Deactivating Kitten reduces the number of eliminated variables

**Fig. 9:**
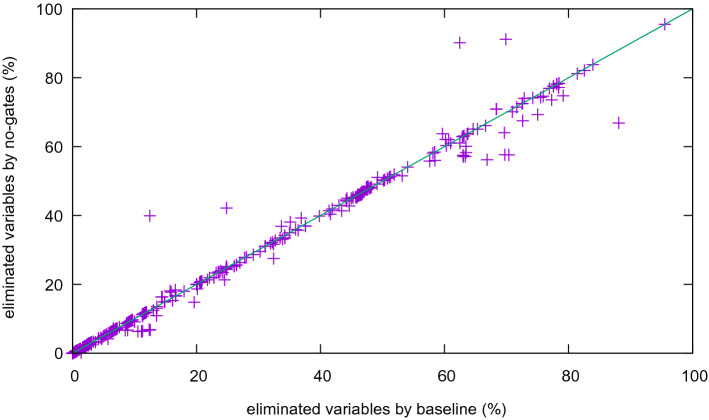
Kissat ’s definition extraction can find gates

**Fig. 10:**
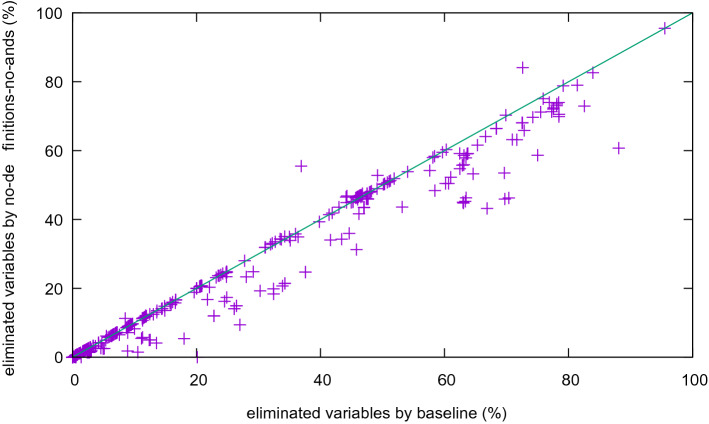
Deactivating And-gate detection leads to fewer eliminated variables

**Fig. 11:**
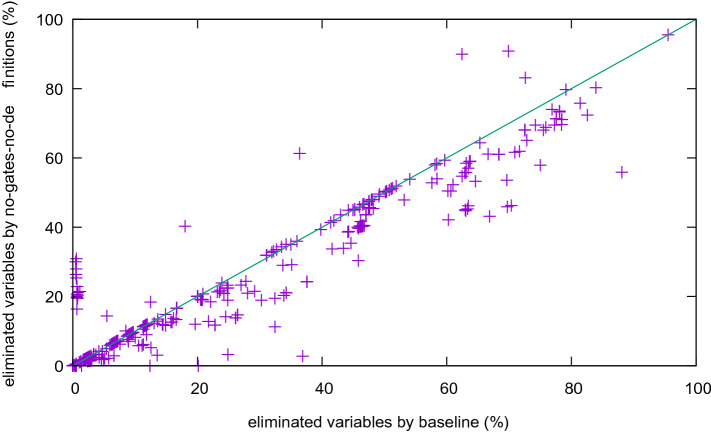
No gate nor definition extraction compared to Kissat ’s base line

We first compare Kissat with definitions and gates (the “base line”) to the version without definitions. To do so, we show the percentage of removed variables in a scatter plot (Fig. 8). More variables are eliminated in the version with definitions. In two extreme cases, more than 90% of the variables are eliminated.

An interesting case is deactivating syntactic extraction of gates^3^ while keeping definition mining through Kitten (Fig. 9). The resulting figure is similar to Fig. 8, indicating that Kitten-based definition mining finds those gates too. Note that Kitten does not necessarily find the minimal (smallest) unsat core, nor is it guaranteed to find a minimum core (an MUS). Thus it could in some cases only find large gates even though small gates exists and thus not eliminate as many variables as possible.

The difference in the number of eliminated variables is much higher if we also deactivate and-gate detection (Fig. 10). With few exceptions the base line removes more variables. Also note that variable elimination is not confluent: eliminating variables in a different order might lead to different results and the number of eliminated variables differs.

Finally, we deactivated syntactic (no-gates) as well as semantic (no-definitions) gate extraction and compare it to the base line (Fig. 11). Much fewer variables are eliminated, as most eliminations need to introduce more clauses.

## Related work

Our approach is mainly motivated by the use of definitions in recent work on model counting [28] and QBF solving [37], where the authors also use core-based techniques, but extract gates explicitly. We showed the connection to this work and claim our restricted formulation is much more concise, because we do not have to extract exactly the variables the definitions depends on.

The approach presented in this article is also the first to use a “little” SAT solver inside a “big” SAT solver to extract definitions, while this related work discussed above uses an ordinary (big) SAT solver to find definitions but for harder problems with a much higher complexity. In circuit synthesis a related approach uses interpolation to find Boolean functions in relations [27].

Another line of work is related to blocked clause elimination [23, 25], a simplification technique used by SAT solvers to remove clauses. A clause is *blocked* if and only if all resolvents with one literal of the clause are tautologies.

Blocked clauses can be removed from the formula, shifting some work from solving (fewer clauses) to model reconstruction (the model after removal might not be a model anymore). However, detecting gates makes it possible to produce fewer clauses even if the solver subsequently uses BCE. Let’s look at the earlier example from Sect. 4:
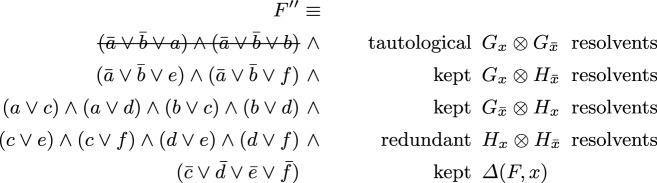


BCE cannot remove the redundant clause $a\vee c$ because it is neither blocked with respect to $a$ (due to clause $\bar{a}\vee \bar{b}\vee e$) nor to $c$ (due to clause $\bar{c}\vee \bar{d}\vee \bar{e}\vee \bar{f}$). By producing fewer clauses during elimination, our method actually makes BCE stronger.

Iser [24] used the “blockedness criterion” to identify gates in addition to a SAT solver (or another approach). He first uses BCE to check that left-uniqueness of the equations, before using the SAT solver to check right-uniqueness. He does not use the SAT solver to identify the clauses, but only to check whether the already identified clauses are right-unique. Iser reports on experiments but does not report on performance changes, only on the amount of time spent in his various strategies.

This work by Iser is also motivated by performing blocked clause decomposition [22], which has the goal to split a CNF in two parts, where the first part is a set of clauses which can be completely eliminated by blocked clause elimination, and the other part contains the remaining clauses. The first “blocked clause set” is of course satisfiable and models can be generated in linear time. This allows to treat that part almost as a circuit [1]. However, blocked clause decomposition is often costly and the second remaining part of clauses often remains big.

## Conclusion

We compute cores with a simple little SAT solver Kitten embedded in a large SAT solver Kissat to semantically find definitions after syntactic gate detection fails in order to eliminate more variables. The cost of calling Kitten is limited by focusing on the environment clauses of elimination candidates and its cheap enough to be used whenever syntactic gate detection fails, while it still allows to produce proofs in the DRAT format when needed.

On the considered benchmark set the performance of Kissat is unfortunately not really improved by semantic definition extraction even though the technique is efficient and effective in finding many additional semantic definitions as well as eliminating more variables. The same applies to syntactic gate detection, which in principle is shown to be subsumed by our new semantic approach.

As future work we want to consider further usage of such an embedded SAT solver and started already to apply it to SAT sweeping [12]. We also want to apply our approach and Kitten to extract definitions for preprocessing in model counting and QBF.

## Footnotes

1 If two clauses can be resolved over two different variables, the resulting resolvents are tautological. Thus the resolution operator “$\otimes$” does not really need to be parameterized by *x*.

2 Resolvents among gate clauses are not necessarily tautological though (see Sect. 8).

3 Using Kissat ’s –no-gate option also deactivates semantic definition extraction. Thus we spelled out all gate types as option in our experiments.
